# Shikonin overcomes drug resistance and induces necroptosis by regulating the miR-92a-1-5p/MLKL axis in chronic myeloid leukemia

**DOI:** 10.18632/aging.103844

**Published:** 2020-09-14

**Authors:** Xianbo Huang, Zhenzhen Chen, Fan Ni, Xiujin Ye, Wenbin Qian

**Affiliations:** 1 Department of Hematology, The First Affiliated Hospital, College of Medicine, Zhejiang University, Hangzhou 310003, China; 2 Department of Hematology, The Second Affiliated Hospital, College of Medicine, Zhejiang University, Hangzhou 310009, China; 3 Department of Hematology, Hangzhou First People’s Hospital, Hangzhou 310006, China; 4 Department of Hematology, The Fourth Affiliated Hospital, College of Medicine, Zhejiang University, Yiwu 322000, China

**Keywords:** chronic myeloid leukemia, shikonin, necroptosis, miR-92a-1-5p, MLKL

## Abstract

Development of resistance to tyrosine kinase inhibitors (TKIs) targeting the BCR/ABL fusion protein represents a major challenge in the treatment of chronic myeloid leukemia (CML). Since apoptosis resistance is the fundamental mechanism impeding TKIs’ therapeutic effects, alternative approaches that induce nonapoptotic cell death are being pursued to treat TKI-resistant CML. Induction of necroptosis, a distinct, caspase-independent form of programmed cell death, may be a valuable strategy in this respect. The present study shows that shikonin, an herbal compound used in traditional Chinese medicine, overcomes TKI resistance in BCR/ABL-positive CML cells by inducing necroptosis via activation of RIPK1/RIPK3/MLKL signaling. This effect occurs both in vitro and in vivo and involves downregulation of miR-92a-1-5p, a poor-prognosis marker frequently overexpressed in leukemia patients. Based on gene expression experiments, we conclude that miR-92a-1-5p promotes CML progression by inhibiting MLKL expression. Accordingly, we show that antagomiR-mediated in vivo inhibition of miR-92a-1-5p reduces the growth of CML tumors in mice through necroptosis induction. Our research suggests that therapies that relieve MLKL suppression by targeting miR-92a-1-5p may represent a useful strategy to treat TKI-refractory CML.

## INTRODUCTION

Chronic myeloid leukemia (CML) is a clonal proliferative neoplasm derived from pluripotent hematopoietic stem cells. CML is characterized by the presence of the Philadelphia chromosome (Ph), which contains a t(9;22)(q34;q11) reciprocal translocation that determines the formation of the chimeric Breakpoint Cluster Region-Abelson (BCR/ABL) gene, which encodes a 210-kDa oncoprotein [1]. The fusion protein product of the *Bcr/Abl* gene is a key factor in CML pathogenesis due to its sustained tyrosine kinase activity, which is able to stimulate numerous oncogenic signaling pathways, including the PI3K/AKT/mTOR, RAS/RAF/MEK/ERK, and JAK/STAT pathways [2]. The advent of tyrosine kinase inhibitors (TKIs), e.g. imatinib, has greatly improved the prognosis and survival of CML patients [3]. However, TKI resistance, which results mainly from BCR/ABL point mutation or gene amplification and also via BCR/ABL-independent mechanisms, is currently a major clinical concern in CML treatment [4]. Second-generation TKIs, including dasatinib and nilotinib, exhibit higher binding affinity for BCR/ABL than imatinib. Although these drugs have appreciably improved the outcomes of patients with imatinib-resistant CML, they are still ineffective against the BCR/ABL gatekeeper mutation T315I [5]. The 3^rd^-generation TKI ponatinib exhibits a favorable anti-CML effect in patients with either wild-type or mutated forms of BCR/ABL, including BCR/ABL-T315I. However, the clinical application of ponatinib is limited by serious side effects, including vascular occlusion, heart failure, and hepatotoxicity [6]. In CML, as well as in many cancers, the development of TKI resistance is commonly associated with resistance to apoptosis [7–9]. Because this represents a major obstacle in clinical oncology, identifying therapies that can induce apoptosis-independent cancer cell death is a pressing necessity [10].

Necroptosis is one of the most well-described caspase-independent, nonapoptotic forms of programmed cell death. Necroptotic cells exhibit necrotic features (early loss of plasma membrane integrity, translucent cytosol, increased cell volume and swollen organelles) that occur, unlike cell necrosis mediated by direct injury, in a highly regulated manner [11]. Increasingly, new research is focusing on necroptosis induction as a potential strategy to eliminate apoptosis-resistant tumor cells [11, 12]. Necroptosis can be commonly triggered by tumor necrosis factor α (TNFα), TNF-related apoptosis-inducing ligand (TRAIL), and Fas ligand (FasL) under apoptosis-deficient conditions [13]. However, several other stimuli, including some kinase inhibitors, viruses, and radiotherapy, can also trigger necroptosis in cancer cells [14]. Insight into the molecular mechanisms of necroptosis has come primarily through the study of TNFα/TNFR (TNF receptor) signaling [13], which revealed the essential role of a ‘necrosome’ complex, formed by receptor-interacting protein kinases 1 and 3 (RIPK1, RIPK3). Within the necrosome, activated RIPK3 recruits and phosphorylates mixed lineage kinase-domain like protein (MLKL), which translocates to and permeabilizes the plasma membrane to execute necroptotic cell death [15].

Shikonin is a naphthoquinone purified from *Lithospermum erythrorhizon*, an herb used in traditional Chinese medicine to treat burns, measles, carbuncles, sore throat, and macular eruptions [16]. Recently, shikonin has been found to exert antitumor effects in several types of cancer (such as osteosarcoma, glioma, breast cancer, and multiple myeloma) by inducing necroptosis via reactive oxygen species (ROS) generation, mitochondrial membrane potential (MMP) loss, and RIPK1/RIPK3 activation [17–20]. However, whether shikonin can induce necroptosis in CML cells (especially TKI-resistant ones) has not been clarified. In this study, we evaluated the antiproliferative effects of shikonin on both TKI-sensitive and TKI-resistant CML cells harboring the *Bcr/Abl* fusion gene and identified, by transcriptomics and reporter expression analyses, an miRNA-based mechanism regulating MLKL expression and necroptosis onset in CML cells.

## RESULTS

### Shikonin reduces CML cell viability in a dose- and time-dependent manner

To evaluate the potential efficacy of shikonin against CML, its cytotoxic effects were first contrasted with those exerted by the TKI imatinib. To this end, CML cells, i.e., human K562 cells and murine myeloid 32D cell lines stably expressing either human wild-type BCR/ABL (32Dp210) or the BCR/ABL T315I mutation (32Dp210-T315I) were treated with different imatinib concentrations for 24 h, and cell viability was then assayed using the MTT assay. As shown in Supplementary Figure 1A, imatinib inhibited K562 and 32Dp210 cell proliferation in a concentration-dependent manner, but had no significant effect on 32Dp210-T315I cells. In contrast, the viability of the three CML cell lines decreased significantly after 3-h exposure to increasing shikonin concentrations (Supplementary Figure 1B). In these experiments, the half maximal inhibitory concentration (IC50) of shikonin for K562, 32Dp210, and 32Dp210-T315I cells was 15.23 μM, 11.17 μM, and 12.48 μM, respectively. Moreover, the survival rate of CML cells incubated with 10 μM shikonin also decreased time dependently, from 1.5 to 24 h (Supplementary Figure 1C). These data indicate that shikonin inhibits CML cell proliferation in a dose- and time-dependent manner and is also effective against imatinib-resistant CML cells.

### Necrostatin-1 blocks shikonin-mediated inhibition of CML cell viability

**Figure 1:**
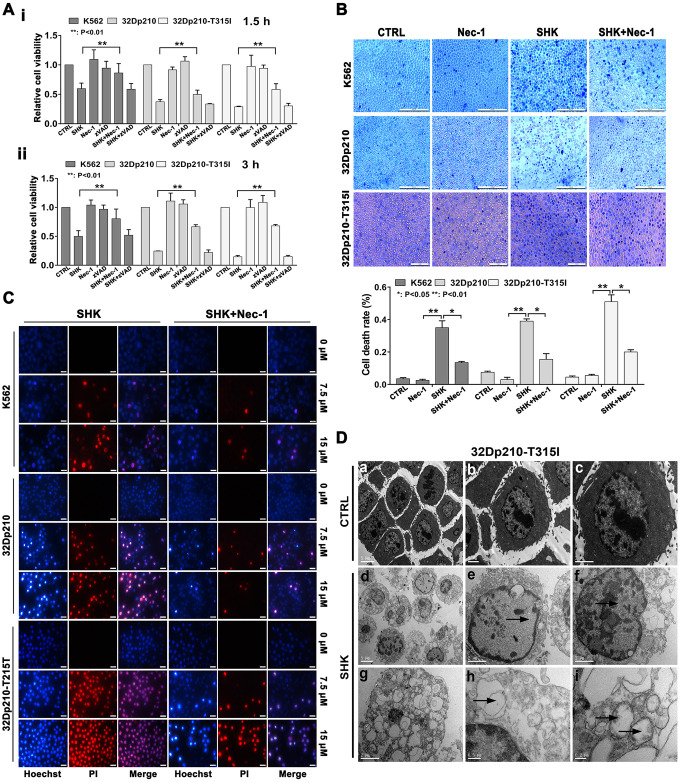
**Shikonin induces necroptosis in CML cells.** (**A**) Results of MTT proliferation assays in K562, 32Dp210, and 32Dp210-T315I CML cells treated with 10 μΜ shikonin for 1.5 h (**i**) or 3 h (**ii**) following pre-treatment (1 h) with Nec-1 (50 μΜ) or zVAD-fmk (35 μΜ). Data are presented as the mean ± SD of three independent experiments. (**B**) Trypan blue exclusion assay results. CML cells were pretreated with or without 50 μΜ Nec-1 for 1 h and subsequently exposed to 10 μΜ shikonin for 3 h. The cells were then stained with trypan blue and the percentage of dead cells was determined under light microscopy. (**C**) Hoechst 33342/PI double staining was performed in CML cells preincubated with or without 50 μΜ Nec-1 and then treated with 0, 7.5, or 15 μΜ shikonin for 3 h. The percentage of PI-permeable cells in each group was determined by fluorescence microscopy. Blue fluorescence indicates staining with Hoechst 33342 and red fluorescence indicates PI staining. A few cells exhibited apoptotic characteristics (chromatin condensation and nuclear fragmentation), as indicated by white arrowheads. Magnification, 200×. (**D**) Electron microscopic examination of 32Dp210-T315I cells treated with 20 μM shikonin for 3 h revealed typical necrotic changes, including disorganization and loss (empty bubble-like formations) of cytoplasmic structures and plasma membrane rupture (**d**, **g**, **h**); syncytial nuclei with chromatin dissolution and disappearance of nucleoli (**e**, **f**); and severe damage to mitochondria with disruption of internal structures (**i**). Scale bars: 5 μm (**a**, **d**), 2 μm (**b**, **c**, **e**, **f**, **g**), 0.5 μm (**h**), and 0.2 μm (**i**). Quantification data are presented as the mean ± SD of three independent experiments. Representative results from three samples are shown. *p < 0.05; **p < 0.01.

To explore the mechanism underlying shikonin-induced CML cell death, apoptosis and necroptosis inhibitors were applied to cell cultures 1 h before shikonin treatment. As shown in Figure 1A, after incubation with shikonin for 1.5 or 3 h in the absence of inhibitors, MTT assays indicated that the survival rate of K562, 32Dp210 and 32Dp210-T315I cells decreased significantly. Relative to this effect, the corresponding survival rates were increased by 15-45% (p < 0.01) after pretreatment with the selective necroptosis inhibitor necrostatin-1 (Nec-1). In contrast, shikonin-induced cell death was not attenuated by pretreatment with zVAD-fmk, a broad-spectrum inhibitor of caspase-dependent apoptosis. The above results indicate that under the above experimental conditions, shikonin-induced death in CLM cells is primarily mediated by necroptosis.

### Morphological characterization of shikonin-induced necroptosis in CML cells

Unlike apoptosis, necroptosis is characterized by disruption of plasma membrane integrity leading to cell swelling and loss of nuclear organization. To verify that these morphological features occur in shikonin-treated CML cells, membrane permeability was first examined by trypan-blue exclusion assays. As shown in Figure 1B, cells in the control group exhibited normal shapes and almost no staining. In contrast, extensive trypan-blue staining was observed in shikonin-treated cells, and this effect was markedly attenuated after preincubation with Nec-1. Further confirmation of shikonin-induced necroptosis was obtained by assessing membrane integrity and nuclear morphology via Hoechst 33342/PI dual staining. Hoechst 33342, a bisbenzimidazole dye, can penetrate intact cells and stain DNA. Due to chromatin aggregation during apoptosis, the resulting fluorescence is much brighter in apoptotic than in nonapoptotic cells [21]. On the other hand, PI incorporation indicates a loss of plasma membrane integrity and can thus be used to distinguish necroptotic cells from normal and early apoptotic cells [22, 23]. As shown in Figure 1C, control CML cells exhibited dark blue, intact nuclei. After shikonin treatment, several nuclei had a normal size but exhibited both dark blue and red fluorescence, indicative of necroptosis. However, a few cells were instead shrunk and had fragmented nuclei exhibiting both blue and red fluorescence (indicated by arrows in Figure 1C), suggesting that they were end-stage apoptotic cells. Interestingly, Nec-1 preincubation significantly reduced the number of necroptotic cells but did not alter the proportion of apoptotic cells. Consequently, we concluded that necroptosis is the main, but not the exclusive, mechanism underlying shikonin-induced CML cell death.

To further characterize the necroptotic changes induced by shikonin, ultrastructural alterations in CML cells were evaluated by transmission electron microscopy (TEM). As shown in Figure 1D, untreated 32Dp210-T315I cells displayed microvilli protruding from the entire surface, a smoothly outlined nucleus with a clearly visible nucleolus, chromatin in the form of heterochromatin, and well-preserved cytoplasmic organelles of high electron density. In contrast, cells treated with shikonin displayed hallmarks of necrotic death, including an electron-lucent cytoplasm, extensive cytoplasmic organelle vesiculation, dilated endoplasmic membranes, cytoskeletal degradation, and plasma membrane rupture with a relatively intact nuclear membrane. In addition, mitochondria were severely damaged, exhibiting swelling, reduced matrix density, and loss of cristae. Also consistent with necroptosis, cell nuclei exhibited obvious swelling and chromatin and nucleolar disaggregation [11, 15].

### Shikonin-induced necroptosis occurs through activation of the RIPK1/RIPK3/MLKL pathway

**Figure 2:**
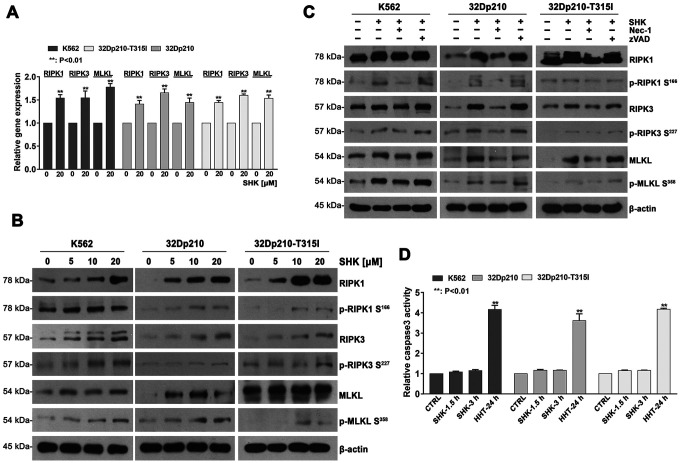
**Shikonin-induced necroptosis is dependent on activation of RIPK1/RIPK3/MLKL signaling.** (**A**) Determination by qRT-PCR of RIPK1, RIPK3 and MLKL expression in CML cells treated with or without 20 mM shikonin for 45 min. Gene expression values were normalized to those of GAPDH. Data represent the mean ± SD of three independent experiments. **p < 0.01. (**B**, **C**) Western blot analysis of both total and phosphorylated RIPK1, RIPK3, and MLKL expression. CML cells were treated with increasing shikonin concentrations (0, 5, 10, or 20 μM) for 45 min without (**B**) or with (**C**) prior exposure to 50 μM Nec-1 or 35 μM zVAD-fmk. (**D**) Apoptosis assessment. CML cells were exposed to 20 μM shikonin for 1.5 or 3 h. Cells treated with 20 nM HHT for 24 h were used as positive control. Apoptosis was measured by a colorimetric assay to detect caspase-3 activation. Data represent the mean ± SD of three independent experiments. **p < 0.01.

RIPK1/RIPK3/MLKL activation is a crucial step in necroptotic signaling pathways [24]. Therefore, we assessed whether shikonin exposure affects the expression and activation of these proteins in CML cells. Our qRT-PCR assays showed that incubation with shikonin for 45 min significantly increased RIPK1, RIPK3, and MLKL mRNA levels in CML cells (p < 0.01, Figure 2A). In turn, western blot analysis suggested that shikonin treatment increased both total expression and the phosphorylation status of the three proteins (Figure 2B). Importantly, Nec-1, but not zVAD-fmk, prevented the activation of RIPK1, RIPK3, and MLKL (Figure 2C). These data indicate that shikonin-induced necroptosis of CML cells proceeds through RIPK1/RIPK3/MLKL pathway activation. To further confirm that shikonin-induced CML cell death is primarily nonapoptotic, a colorimetric assay was used to evaluate caspase-3 activation. As positive control we used homoharringtonine (HHT), which was shown to induce apoptosis in CML cells [25]. As shown in Figure 2D, caspase-3 activation was only observed in CML cells treated with HHT (p < 0.01). These data confirm that shikonin induces caspase-independent necroptosis in CML cells.

### Shikonin-induced necroptosis determines ROS overproduction, MMP loss, and inactivation of the BCR/ABL oncoprotein

**Figure 3:**
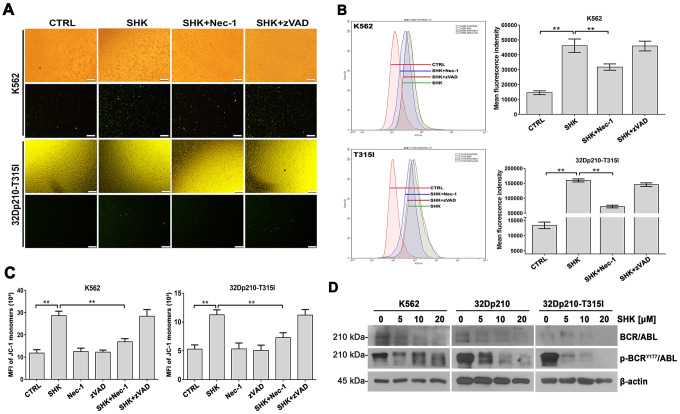
**Shikonin induces ROS production and MMP loss in CML cells.** (**A**, **B**) Assessment of ROS generation by H_2_DCFDA staining. K562 and 32Dp210-T315I cells were pretreated with or without Nec-1 (50 μM) or zVAD-fmk (35 μM) for 1 h and then exposed to 20 μM shikonin for 1 h. Upper row images in (**A**) are phase contrast micrographs, and lower row images are fluorescence micrographs. Green signal indicates ROS generation as detected by H_2_DCFDA staining. Quantitation of DCF fluorescence from flow cytometry assays is shown in (**B**). Values are expressed as the mean ± SD of 3 experiments. **p < 0.01. (**C**) Analysis of MMP by JC-1 staining. Control, Nec-1 (50 μM), or zVAD-fmk (35 μM) pre-treated cells were exposed to 20 μM shikonin for 1 h, stained with JC-1 dye, and analyzed by flow cytometry. Results represent data from three independent experiments. (**D**) Western blot analysis of total and phosphorylated BCR/ABL expression in CML cells treated with 5-20 μM shikonin for 3 h.

ROS production has been suggested to drive TNFα-mediated necroptosis in some cell types [26, 27]. To evaluate whether this mechanism also underlies shikonin-induced CML cell death, we examined ROS levels using fluorescence microscopy and flow cytometry after loading cells with the redox-sensitive dye H_2_DCFDA. The fluorescence images in Figure 3A indicate that shikonin treatment led to intracellular ROS accumulation in CML cells, which was markedly inhibited in the presence of Nec-1 but not zVAD-fmk. This observation was confirmed by quantification of the fluorescence intensity under each experimental condition (Figure 3B). These data suggest that shikonin-induced necroptosis was associated with ROS overproduction in CML cells.

Since excessive ROS production is eminently linked to mitochondrial dysfunction, we next loaded CML cells with the potentiometric dye JC-1 and determined by flow cytometry whether alterations in mitochondrial membrane potential (MMP) occur during shikonin-mediated necroptosis. As shown in Figure 3C, a significant increase in green fluorescence was detected after shikonin exposure. This implied a prevalence of monomeric JC-1, indicative of MMP loss. In contrast, the number of cells with depolarized MMP was significantly reduced after Nec-1 but not zVAD-fmk pretreatment. Therefore, we concluded that shikonin-induced necroptosis is closely related to mitochondrial dysfunction in CML cells.

Although previous studies have demonstrated apoptosis in BCR/ABL-positive CML cells (e.g., K562 cells) treated with low shikonin concentrations (0.2-1 μM) [28], the effects of shikonin on the BCR/ABL signaling pathway have not yet been reported. Using western blotting, we detected dose-dependent inhibition of BCR/ABL expression in CML cell lines treated with higher shikonin concentrations (5-20 μM). Moreover, this effect was accompanied by a concomitant reduction in BCR/ABL phosphorylation at Tyr177 (Figure 3D). These data indicate that shikonin effectively inhibits BCR/ABL activity in CML cells.

### Shikonin inhibits CML tumorigenesis in vivo

**Figure 4:**
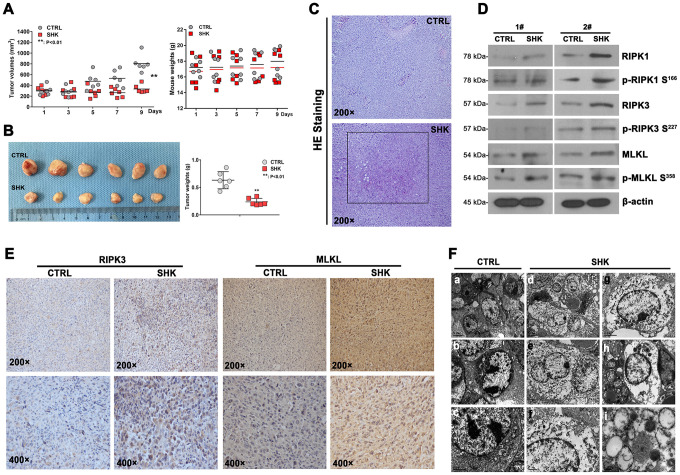
**Shikonin inhibits the growth of TKI-resistant CML cells in vivo.** (**A**) Quantification of mouse weights and mean tumor volumes. NOD/SCID mice were subcutaneously implanted with 32Dp210-T315I cells and treated i.p. with shikonin or PBS (as control) daily for 9 days (n = 6 mice/group). Data are presented as mean ± SD. **p < 0.01. (**B**) Macroscopic appearance and mean weight of excised 32Dp210-T315I tumors. **p < 0.01. (**C**) HE analysis showing distinct necrotic features in tumor samples from shikonin-treated mice. (**D**) Western blotting analysis of total and phosphorylated RIPK1, RIPK3, and MLKL expression in 32Dp210-T315I tumor samples. (**E**) Immunohistochemical analysis of RIPK3 and MLKL expression in tumor tissues. (**F**) Morphological analysis of tumor tissues via electron microscopy. Bars: 5 μm (**a** and **d**), 2 μm (**b**, **c**, **e**, **g**, and **h**), 1 μm (**f**), and 0.2 μm (**i**).

To evaluate the ability of shikonin to inhibit CML development in vivo, murine 32Dp210-T315I cells were grafted into NOD/SCID mice by subcutaneous injection. After 7 days, tumor-bearing mice were intraperitoneally injected with shikonin (3 mg/kg·d or PBS (n = 6 mice/group) daily for a total of 9 injections. The mice were euthanized two days after the last injection, and mouse weights and tumor volumes were then determined. The mouse weights were not significantly different between treatment groups (18.0 ± 1.8 g vs 17.2 ± 2.0 g; Figure 4A). Importantly, the mean tumor volume was significantly lower in shikonin-treated mice than in control mice (332.7 ± 76.0 mm^3^ vs 808.3 ± 156.9 mm^3^; p < 0.01; Figure 4A). After excision, the tumors of shikonin-treated mice were smaller, paler, and weighed significantly less than those of control mice (0.237 ± 0.065 g vs 0.631 ± 0.156 g; p < 0.01; Figure 4B). These results indicate that shikonin inhibits angiogenesis and growth of CML grafts harboring T315I-mutant BCR/ABL kinase. Consistent with necroptosis, both necrosis (Figure 4C) and activation of the RIPK1/RIPK3/MLKL pathway (Figure 4D) were observed in tumors from shikonin-treated mice. The latter western blot data were in turn supported by immunohistochemical analyses that showed increased RIPK3 and MLKL expression in these samples (Figure 4E). Moreover, similar to the above in vitro findings, morphological characteristics consistent with necroptosis were revealed by TEM imaging (Figure 4F). These results indicate that shikonin has strong anti-CML effects in vivo that are also mediated by necroptotic cell death.

### Shikonin induces miRNA transcriptome alterations in CML cells

**Figure 5:**
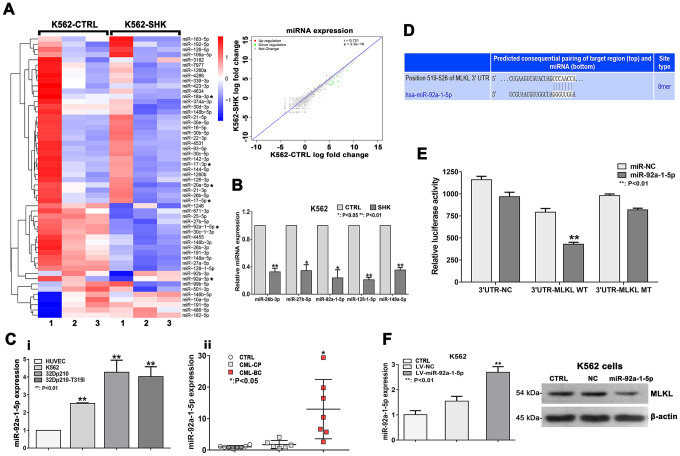
**Downregulation of miR-92a-1-5p, a negative MLKL regulator, mediates shikonin-induced necroptosis in CML cells.** (**A**) Heat map of miRNA expression profiles from control (PBS-treated) and shikonin-treated (20 μM, 1 h) K562 cells. The expression of each miRNA corresponds to the average of 3 replicates. Asterisks indicate miRNAs in the miR-17-92 cluster. Also shown is the correlation of the log fold expression changes of the 1,342 miRNAs with differential expression between the two groups. For the shikonin group, transcripts with >2-fold decrease in are shown in green, while those with >2-fold increase are shown in red. (**B**) Validation of NGS data by miRNA-specific qRT-PCR. Values are presented as the mean ± SD of 3 independent experiments. *p < 0.05; **p < 0.01. (**C**) Analysis of relative miR-92a-1-5p expression in CML cell lines and HUVECs (**i**); Relative miR-92a-1-5p expression in CML patient samples (**ii**). (**D**) Sequence alignment of the 3’UTR of human (hsa) MLKL mRNA, highlighting an 8-mer binding site for miR-92a-1-5p. (**E**) Dual-luciferase reporter assay demonstrating the interaction between miR-92a-1-5p and MLKL mRNA. HEK-293T cells were co-transfected with 50 nM miR-92a-1-5p mimics or NC-mimics and a dual-luciferase vector containing either the wild-type (WT) or mutant (MT) 3’UTR of MLKL. Relative firefly luciferase activity (normalized to Renilla luciferase activity) was determined 48 h after transfection. Data are presented as the mean ± SD of three independent experiments. **p < 0.01. (**F**) qRT-PCR analysis of miR-92a-1-5p expression in K562 cells transduced with lentiviral vectors encoding miR-92a-1-5p. Western blot images exemplify the decrease in MLKL expression induced by miR-92a-1-5p overexpression. All experiments were repeated at least three times.

Since necroptosis induction can be regulated by miRNA expression changes [29], we next conducted NGS to analyze possible shikonin-induced alterations in the miRNA transcriptome of CML cells. To this end, K562 cells were either treated with PBS (control) or exposed to 20 μM shikonin for 1 h prior to collection of total RNA. Saturation analysis indicated that a sequencing depth of approximately 20 million reads per sample was adequate to analyze the miRNA transcriptome (Supplementary Figure 1D, 1E). Reads of 20-24 nt were used for identification and quantification of the miRNA transcriptome. In total, 1527 unique miRNA sequences were detected, represented by 114,465,232 effective read counts. A total of 185 sequences represented candidate unknown mature miRNAs, and 1,342 sequences corresponded to known mature miRNAs included in miRbase. Among the 1,342 known mature miRNAs, 54 were significantly differentially expressed in shikonin-treated cells relative to untreated, control cells (FDR < 0.05). Of those 54 miRNAs, 9 were expressed at higher levels in the shikonin group, while 45 were expressed at lower levels. Interestingly, 6 of these 54 miRNAs (miR-92a-3p, miR-92a-1-5p, miR-20a-5p, miR-18a-3p, miR-17-5p, and miR-17-3p) belong to the miR-17-92 polycistronic cluster, while another transcript, miR-106a-5p, is located in a miR-17-92 paralog cluster [30]. In addition, the expression of 15 miRNAs, namely, miR-92a-1-5p, miR-7977, miR-4634, miR-4531, miR-4455, miR-4286, miR-3182, miR-30c-1-3p, miR-27b-5p, miR-27a-5p, miR-26b-3p, miR-191-3p, miR-148a-5p, miR-128-1-5p, and miR-106a-5p, showed >2-fold change (Figure 5A). NGS data were next validated using miRNA-qRT-PCR. As shown in Figure 5B, this analysis confirmed differential expression of 6 of the 15 most meaningful miRNAs identified by NGS.

We next focused on miR-92a-1-5p, which was the most significantly differentially expressed (downregulated) miRNA in shikonin-treated cells. qRT-PCR analysis showed significantly higher baseline miR-92a-1-5p expression in CML cell lines than in HUVECs (used as a control) (Figure 5Ci). To corroborate these findings, we used qRT-PCR to measure miR-92a-1-5p expression in bone marrow samples from CML patients and from patients with malignant nonhematological disease (selected as controls). Suggesting that miR-92a-1-5p is a putative tumor-promoting factor in CML, higher miR-92a-1-5p expression was detected in CML patients (Figure 5Cii). The clinical characteristics of the CML patients are listed in Supplementary Table 1.

### miR-92a-1-5p directly targets MLKL

To investigate the functional significance of miR-92a-1-5p upregulation in CML, we performed target prediction analysis on the TargetScan search tool to identify candidate miR-92a-1-5p targets among key necroptosis-associated mRNAs. Sequence alignment of the 3’UTR of the MLKL mRNA identified an 8-mer (CCCAACCA) as a putative miR-92a-1-5p binding site (Figure 5D). This interaction was subsequently tested by a dual-luciferase reporter assay in HEK-293T cells. The results showed a significant decrease in MLKL-3’UTR activity upon transfection with a miR-92a-1-5p mimic but not with a scrambled control miRNA. Confirming that MLKL is a direct target of miR-92a-1-5p, no changes in luciferase activity were detected when a mutant MLKL-3’UTR was assayed (Figure 5E). Furthermore, as shown in Figure 5F, lentivirus-mediated overexpression of miR-92a-1-5p in K562 cells resulted in a significant decrease in MLKL expression. These data demonstrate that MLKL is a bona fide target of miR-92a-1-5p.

### Downregulation of miR-92a-1-5p inhibits CML growth in vivo

**Figure 6:**
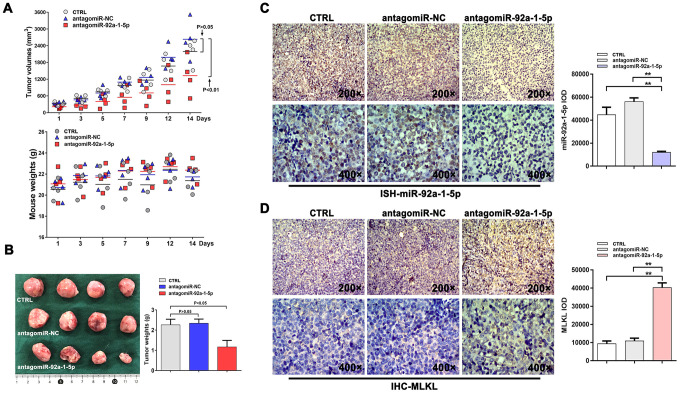
**AntagomiR-mediated inhibition of miR-92a-1-5p in vivo reduces CML xenograft growth.** (**A**) K562 xenografts were established in NOD/SCID mice injected intratumorally with antagomiR-92a-1-5p (8 nmol/d, 25 μL), antagomiR-NC (8 nmol/d, 25 μL), or PBS (25 μL/d, as control) every other day for 2 weeks (n = 4 mice/group). Tumor volumes and mouse weights were measured at the indicated time points. Data are presented as the mean ± SD. **p < 0.01. (**B**) Photographs of excised K562 tumors and corresponding weight measurements. **p < 0.01. (**C**) ISH detection of miR-92a-1-5p expression in excised tumors and integrated optical density (IOD) quantification (mean ± SD; **p < 0.01). (**D**) IHC analysis of MLKL expression in excised tumors and IOD quantification (mean ± SD; **p < 0.01).

Based on the above data, we evaluated the role of miR-92a-1-5p in CML tumorigenesis in NOD/SCID mice. Seven days after subcutaneous inoculation of human K562 cells, the mice received 7 intratumoral injections, every 2 days, of antagomiR-92a-1-5p, antagomiR-NC (negative control), or PBS. Tumor growth inhibition was obvious after the second antagomiR-92a-1-5p injection and continued until sacrifice, i.e., two days after the last injection. As shown in Figure 6A, at this time point, the mean tumor volume was significantly reduced in antagomiR-92a-1-5p-treated mice (V_PBS_ = 2191.0 ± 467.9 mm^3^, V_antagomiR-NC_ = 2632.9 ± 692.5 mm^3^, V_antagomiR-92a-1-5p_ = 1327.1 ± 689.2 mm^3^; n = 4 mice/group). Throughout the experimental period the treatments were well tolerated, and the animals exhibited no behavioral changes, had similar food/water intake levels, and maintained comparable weights (Figure 6A). Following excision, the tumors of mice in the antagomiR-92a-1-5p group were smaller (Figure 6B) and weighed significantly less (1.169 ± 0.643 g) than the tumors from mice in the antagomiR-NC group (2.335 ± 0.423 g) and the PBS group (2.258 ± 0.565 g) (p < 0.05; Figure 6B). Tumor samples were then processed for HE staining, which showed small and discrete necrotic foci as well as accumulation of mononuclear inflammatory cells in tumors that received antagomiR-92a-1-5p (Figure 6C). Importantly, in situ hybridization (ISH) experiments using specific locked nucleic acid (LNA)-modified DIG-labeled probes showed a significant reduction in miR-92a-1-5p expression in antagomiR-92a-1-5p-treated tumors (Figure 6D) compared with the other groups. On the other hand, immunohistochemistry (IHC) showed that MLKL expression was significantly increased in tumors treated with antagomiR-92a-1-5p. These in vivo observations strongly suggest that miR-92a-1-5p has an oncogenic effect on CML through inhibition of the necroptosis executor MLKL.

## DISCUSSION

Drug resistance and metabolic reprogramming are common features of tumor cells [31]. The most aggressive and treatment-resistant form of CML is the subtype harboring the *Bcr/Abl* gene with the T315I mutation, which is resistant to apoptosis induced by nearly all TKIs [1, 5]. Hence, alternative therapies that induce apoptosis-independent cell death are needed to effectively treat patients with this CML variant. In this regard, increasing evidence suggests that anticancer agents that induce necroptosis instead of apoptosis may markedly improve the outcomes of patients with TKI-resistant cancers [12, 32]. Shikonin, an herbal compound used in traditional Chinese medicine, has been reported to induce, through diverse mechanisms, either apoptosis or necroptosis in various tumor cells [17–20]. However, the efficacy of shikonin against TKI-resistant CML has not been explored.

In this study, we demonstrated the ability of shikonin to induce necroptosis both in TKI-sensitive CML cell lines and in TKI-resistant CML cells bearing the T315I mutation in the *Bcr/Abl* gene. In cultured CML cells as well as in 32Dp210-T315I tumors in vivo, shikonin-induced CML cell death could be largely rescued by Nec-1, a selective RIP1 kinase inhibitor that blocks necroptosis, but not by apoptosis in different cells [33]. In contrast, zVAD-fmk, a broad-spectrum apoptosis inhibitor [34], had no obvious protective effect against shikonin-induced CML cell death. Using several assays, we confirmed that the morphological alterations occurring in shikonin-treated CML cells reflected necroptosis rather than apoptosis. The alterations observed included compromised plasma membrane integrity, as determined by trypan blue exclusion assays and evidence of double-positive Hoechst 33342/PI staining, which is frequently used to distinguish necrosis from apoptosis [21, 22]. Moreover, several ultrastructural alterations consistent with necroptosis were also detected in shikonin-treated cells imaged by TEM [24]. Still, a minority of cells displayed features of apoptosis following shikonin treatment. This observation agrees with past research that reported apoptosis of CML cells after exposure to low micromolar shikonin doses and suggests that both dose- and tumor-dependent mechanisms are responsible for shikonin’s wide anticancer effects [28].

RIPK1, RIPK3, and MLKL are considered crucial modulators of necroptosis [15]. Consistent with necroptosis induction, we found that both the expression and the phosphorylation status of these proteins increased in a concentration-dependent manner in shikonin-treated CML cells. Accordingly, RIPK1/RIPK3/MLKL pathway activation was significantly attenuated by Nec-1 but not by zVAD-fmk, and no changes in cleaved caspase-3 levels were observed in these experiments. Recent studies have showed that MMP loss secondary to impaired mitochondrial membrane integrity leads to excessive ROS production and triggers necroptosis in various tumor cells [24, 35]. Similarly, we detected a significant increase in cellular ROS levels as well as a loss of MMP in shikonin-treated CML cells. Since MMP depolarization was largely prevented by Nec-1 but not by zVAD-fmk, we speculated that mitochondrial dysfunction mediates shikonin-induced necroptosis in CML cells. Moreover, western blot analysis showed that treatment with shikonin caused significant reductions in both total and phosphorylated BCR/ABL oncoprotein levels. Although further research is warranted, these changes may represent secondary necroptotic events. Importantly, we showed that systemic (intraperitoneal) shikonin therapy significantly attenuated the growth of CML tumors formed by 32Dp210-T315I cells. Consistent with our in vitro findings, excised tumors showed upregulation and activation (phosphorylation) of RIPK1/RIPK3/MLKL and morphological changes indicative of necroptosis. These results suggest that shikonin might be effective to overcome TKI-resistance in BCR/ABL-T315I CML.

Studies have shown that necroptosis can be regulated by several miRNAs (e.g. miR-155, miR-499, miR-19, and miR-181) through direct or indirect regulation of the expression of necroptosis-related proteins [29]. Using NGS, we obtained miRNA expression profiles in shikonin-treated K562 CML cells and identified 54 differentially expressed miRNAs. Among these were 6 members of the polycistronic miR-17-92 cluster, which are usually overexpressed and act as tumor promoters in a BCR/ABL- and c-myc-dependent manner in CML [36–38]. Thus, we speculated that miR-17-92 transcripts may participate in the posttranscriptional regulation of key necroptosis genes. Among those transcripts, miR-92a-1-5p showed the most significant expression change. Suggesting a key role in CML progression, miR-92a-1-5p was highly expressed in both untreated CML cell lines and clinical CML samples and markedly downregulated after shikonin exposure in K562 cells. Recent studies have shown that miR-92a-1-5p expression is frequently increased in acute myelocytic leukemia (AML) and acute lymphocytic leukemia (ALL) and indicates a poor prognosis [39]. However, the effect of dysregulated miR-92a-1-5p expression in CML and its impact on necroptosis induction have not been clarified. Our dual-luciferase assay results proved that MLKL, a critical mediator of necroptosis [40, 41], is a direct target of miR-92a-1-5p. Accordingly, we showed that miR-92a-1-5p overexpression led to a decrease in MLKL expression and stimulated proliferation in cultured K562 cells. Giving strong support to the above in vitro evidence, we showed that a specific miR-92a-1-5p antagonist inhibits the growth of K562 xenografts in mice. These data strongly suggest that miR-92a-1-5p upregulation promotes proliferation and survival of CML cells by downregulating MLKL expression.

In summary, our study provides solid evidence that miR-92a-1-5p promotes CML progression by inhibiting MLKL expression and activity. Notably, exposure to shikonin downregulates miR-92a-1-5p and induces necroptosis via activation of RIPK1/RIPK3/MLKL signaling in both TKI-sensitive and TKI-resistant CML cells. These findings indicate that necroptosis induction via shikonin or other agents targeting miR-92a-1-5p may represent a novel therapeutic strategy to treat apoptosis-resistant CML [15].

## MATERIALS AND METHODS

### Drugs and reagents

Shikonin was purchased from Sigma (St. Louis, MO, USA). The broad-spectrum caspase inhibitor zVAD-fmk and the TKI imatinib were obtained from Selleck Chemicals (Houston, TX, USA). Nec-1 was purchased from Calbiochem (Cambridge, MA, USA). H_2_DCFDA and JC-1 were obtained from Molecular Probes (Eugene, OR, USA). All these agents were dissolved in dimethyl sulfoxide (DMSO) at suitable storage concentrations according to the manufacturers’ instructions.

### Cell lines and cell culture

Human K562, HEK-293T, and HUVEC cell lines and murine leukemic 32D cells with wild-type BCR/ABL (32Dp210) or T315I mutant BCR/ABL (32Dp210-T315I) were obtained from the Institute of Hematology, Zhejiang University (Hangzhou, China). The CML cell lines were grown in RPMI-1640 medium (HyClone Laboratories, Logan, UT, USA). HUVECs and HEK-293T cells were cultured in DMEM (HyClone Laboratories). All cultures were supplemented with 10% heat-inactivated fetal bovine serum (Gibco) and penicillin/streptomycin (100 U/mL penicillin and 100 μg/mL streptomycin, Sigma) and maintained at 37 °C in a humidified atmosphere containing 5% CO_2_.

### Primary CML cells

Human primary CML cells were obtained from CML patients undergoing bone marrow puncture, while bone marrow control samples were collected from patients with malignant nonhematological disease. The samples were collected between March 2017 and August 2017 at the First Affiliated Hospital, College of Medicine, Zhejiang University (China) after informed consent and ethics committee approval were obtained. Bone marrow samples were subjected to total leukocyte separation and frozen in liquid nitrogen until use.

### Cell viability assay

K562 (2 x 10^4^ cells/well), 32Dp210 (4 x 10^4^ cells/well), and 32Dp210-T315I (4 x 10^4^ cells/well) CML cells were seeded in 96-well plates and treated with PBS (control) or shikonin as indicated. Cell viability was determined by incubation with fresh medium containing 0.5 mg/mL MTT [3-(4,5-dimethylthiazol-2-yl)-2,5-diphenyl tetrazolium bromide; Sigma] for 4 h at 37 °C. The MTT-containing medium was then removed and DMSO (200 μL) was added to each well. Well absorbance was measured at 570 nm in a microplate spectrophotometer (Bio-Rad, Richmond, CA, USA).

### Trypan blue exclusion assay and Hoechst/PI staining

CML cells were cultured at a density of 1×10^5^/mL in a 6-well plate and treated for 1 h with or without 50 μM Nec-1 or 30 μM zVAD-fmk prior to 3-h exposure to shikonin. Cell pellets were collected, washed, and resuspended in PBS, and plasma membrane integrity was determined by trypan blue (0.4%) staining (Sigma) using light transmission microscopy. To identify cells with nuclear changes typical of apoptosis or necroptosis, collected cells were successively incubated with 1 mg/mL Hoechst 33342 (Sigma) and 10 μg/mL PI (MultiSciences Biotech, China) for 10 min at room temperature and observed under a fluorescence microscope (Leica Microsystems, Wetzlar, Germany). An excitation wavelength of 450-490 nm was used to visualize PI, and 330-380 nm was used to visualize Hoechst 33342. At least three random microscopic fields per sample were imaged. The experiments were repeated at least three times.

### Transmission electron microscopy

TEM imaging was conducted in cultured cells and tumor samples. Cultured cells were washed twice with PBS and fixed overnight with 2.5% glutaraldehyde in PBS (pH 7.4). Tumors excised from NOD/SCID mice were cut into 1 mm^3^ pieces and fixed overnight with 2.5% glutaraldehyde solution. The samples were treated with 1.0% osmium tetroxide, dehydrated through a graded ethanol series (50%, 70%, 90%, and 100%), and embedded in Durcupan resin. Ultrathin sections (65 nm) were sliced with an ultramicrotome, poststained with 1% uranyl acetate and 0.1% lead citrate and examined using a TECNAI 10 electron microscope (Philips Electronic Instruments, Holland) at 60 kV.

### qRT-PCR assay

Total RNA extraction was carried out with RNAiso Plus (Takara Shuzo, Kyoto, Japan). cDNA templates were generated from total RNA using an M-MLV reverse transcriptase kit (Invitrogen, Carlsbad, CA, USA) according to the manufacturer’s instructions. The sequences of the mRNA and miRNA primers used to amplify the genes of interest are listed in Table 1 and Table 2, respectively.

**Table 1:** mRNA primer sequences.

**Name**	**Forward**	**Reverse**
GAPDH (Human)	5’-AAGGTGAAGGTCGGAGTCA-3’	5’-GGAAGATGGTGATGGGATTT-3’
GAPDH (Mouse)	5’-AGCAGTCCCGTACACTGGCAAAC-3’	5’-TCTGTGGTGATGTAAATGTCCTCT-3’
RIPK1 (Human)	5’-CGTAAACTGGGCTTCACACA-3’	5’-TTATGCCTTCCCTCATCACC-3’
RIPK1 (Mouse)	5’-GCAGGAGCAAGAGGTCATTC-3’	5’-TGGCTTAGATTTGGCGGATA-3’
RIPK3 (Human)	5’-ATGAATGCTGCTGTCTCCAC-3’	5’-TGGTTCTCCTAAAGCCATCC-3’
RIPK3 (Mouse)	5’-GGCTCTCGTCTTCAACAACTG-3’	5’-CCGAACTGTGCTTGGTCATA-3’
MLKL (Human)	5’-GGAGGCTAATGGGGAGATAGA-3’	5’-TCCTTCCAGACATCACTCAGC-3’
MLKL (Mouse)	5’-CCCATTTGAAGGCTGTGATT-3’	5’-ATGATTTCCCGCAACAACTC-3’

**Table 2:** miRNA primer sequences.

**Name**	**Primer**	**Sequence**
U6	RT primer	5’-CGCTTCACGAATTTGCGTGTCAT-3’
U6	Forward	5’-GCTTCGGCAGCACATATACTAAAAT-3’
U6	Reverse	5’-CGCTTCACGAATTTGCGTGTCAT-3’
miR-92a-1-5p (Human)	RT primer	5’-GTCGTATCCAGTGCGTGTCGTGGAGTCGGCAA-
		TTGCACTGGATACGACAGCATTGC-3’
miR-92a-1-5p (Human)	Forward	5’-AGGTTGGGATCGGTTGC-3’
miR-92a-1-5p (Human)	Reverse	5’-CAGTGCGTGTCGTGGAGT-3’
miR-128-1-5p	RT primer	5’-GTCGTATCCAGTGCGTGTCGTGGAGTCGGCAA-
		TTGCACTGGATACGACTCTCAGAC-3’
miR-128-1-5p	Forward primer	5’-CGGGGCCGTAGCACTGT-3’
miR-128-1-5p	Reverse primer	5’-CAGTGCGTGTCGTGGAGT-3’
miR-148a-5p	RT primer	5’-GTCGTATCCAGTGCGTGTCGTGGAGTCGGCAA-
		TTGCACTGGATACGACAGTCGGAG-3’
miR-148a-5p	Forward primer	5’-AAAGTTCTGAGACACTC-3’
miR-148a-5p	Reverse primer	5’-CAGTGCGTGTCGTGGAGT-3’
miR-26b-3p	RT primer	5’-GTCGTATCCAGTGCGTGTCGTGGAGTCGGCAA-
		TTGCACTGGATACGACGAGCCAAG-3’
miR-26b-3p	Forward primer	5’-CCTGTTCTCCATTACT-3’
miR-26b-3p	Reverse primer	5’-CAGTGCGTGTCGTGGAGT-3’
miR-27b-5p	RT primer	5’-GTCGTATCCAGTGCGTGTCGTGGAGTCGGCAA-
		TTGCACTGGATACGACGTTCACCA-3’
miR-27b-5p	Forward primer	5’-AGAGCTTAGCTGATTG-3’
miR-27b-5p	Reverse primer	5’-CAGTGCGTGTCGTGGAGT-3’

### Western blot analysis

Cultured cells were lysed at 4 °C in lysis buffer and protein concentrations determined by the bicinchoninic acid (BCA) method. Equal amounts of total cell lysates were separated on sodium dodecyl sulfate (SDS)-polyacrylamide gels (8-12% gradient) and transferred to PVDF membranes (Millipore, Bedford, MA, USA). Membranes were blocked for 2 h with Tris-buffered saline containing 0.1% Tween and 5% nonfat dry milk and incubated with primary antibodies overnight at 4 °C. After incubation with horseradish peroxidase-conjugated secondary antibodies (1:5000; Multi Sciences Biotech), immunoreactions were visualized with an ECL detection kit (Biological Industries, Beit HaEmek, Israel). Primary antibodies specific for the following proteins were used: RIPK1, p-RIPK1 (Ser166), BCR/ABL, p-BCR/ABL (Tyr177), and β-actin (Cell Signaling Technology, CST; Beverly, MA, USA); RIPK3, p-RIPK3 (Ser223), MLKL, and p-MLKL (Ser358) (Abcam, Cambridge, UK).

### Detection of caspase-3 activity

CML cells were seeded in 12-well plates (1×10^5^ cells/well) and exposed to shikonin (20 μM) for 3 h. Cells treated with 20 nM HHT (Sigma) were used as positive control. Caspase-3 activity was measured using a caspase-3/CPP32 colorimetric assay kit (BioVision, Mountain View, CA) according to the manufacturer’s instructions. Sample absorbance was determined at 405 nm with a microplate spectrophotometer (Bio-Rad) and is expressed as fold increase relative to control (PBS-treated cells).

### Measurement of intracellular ROS levels

The redox-sensitive dye H_2_DCFDA (Molecular Probes) was used to measure intracellular ROS generation in K562 and 32Dp210-T315I CML cells 1 h after treatment with shikonin, either alone or following pretreatment with 50 μM Nec-1 or 35 μM zVAD-fmk. To this end, the cells were centrifuged at 1500 rpm for 5 min, washed twice with PBS, and loaded with 1 μM H_2_DCFDA in the dark at 37 °C for 15 min. Following resuspension in 1% Triton X-100, 2’,7’-dichlorofluorescein (DCF) fluorescence was detected at 485/530 excitation/emission wavelengths via fluorescence microscopy and flow cytometry.

### Analysis of mitochondrial membrane potential (MMP)

The potentiometric probe JC-1 (Molecular Probes) was used for MMP measurements according to the manufacturer’s instructions. JC-1 molecules are uptaken and oligomerize (visualized as red fluorescence) in polarized mitochondria and are released as monomers (visualized as green fluorescence) upon MMP loss. Briefly, CML cells subjected to different treatments were collected, centrifuged, washed with PBS, and stained with 2 μg/mL JC-1 in PBS for 10 min at 37 °C. Green fluorescence was measured by flow cytometry.

### CML tumor models and treatment regimens

All animal experiments were reviewed and approved by the Animal Care and Use Committee of the First Affiliated Hospital, College of Medicine, Zhejiang University. Human K562 and murine 32Dp210-T315I CML cell lines were cultured under the standard conditions described above. Prior to implantation, cells were harvested and resuspended in sterile PBS at 1×10^8^ cells/mL. NOD/SCID mice (Shanghai Experimental Animal Center of the Chinese Academy of Sciences, Shanghai, China) were housed in IVC cages under SPF conditions. A 100 μL aliquot of the corresponding cell suspension (1×10^7^ cells per animal) was injected subcutaneously into the right flank of 4-week-old male NOD/SCID mice. On day 7 after inoculation the mice implanted with 32Dp210-T315I cells were divided into two groups (n = 6 mice/group) and administered, respectively, shikonin (3 mg/kg·d, 200 μL) or PBS (200 μL) via intraperitoneal (i.p.) injection (once daily for 9 days). In turn, the mice implanted with K562 cells were separated into three groups (n = 4 mice/group) and administered, respectively, antagomiR-92a-1-5p (8 nmol/d, 25 μL), antagomiR-NC (8 nmol/d, 25 μL), or PBS (25 μL) via intratumoral injection, every other day for a total of 7 injections. Tumor volumes and mouse weights were measured during the experiment. Mice were euthanized two days after the last injection. The tumors were then excised, weighed, and subjected to further analyses.

### Histopathological and immunohistochemical analyses

Grafted tumors were fixed in 4% paraformaldehyde, embedded in paraffin, and sliced into 4-μm sections. The degree of necrosis was evaluated by HE staining. RIPK3 and MLKL were detected in tumor samples by immunohistochemistry using specific antibodies. Some samples were also processed for TEM imaging as described above.

### miRNA transcriptome sequencing

K562 cells were treated with PBS (control) or shikonin (20 μM for 1 h) and duplicates of 3 control samples (K562-control1, K562-control2, K562-control3) and 3 test samples (K562-shikonin1, K562-shikonin2, K562-shikonin3) were processed for miRNA sequencing. Total RNA was isolated with a miRNeasy Mini kit (Qiagen) according to the manufacturer’s instructions and sequenced on an Illumina HiSeq platform.

### Vector construction and dual-luciferase reporter assay

The 3’UTR of MLKL mRNA containing miR-92a-1-5p binding sites was cloned into the PGL3-CMV-LUC-MCS vector downstream of the luciferase reporter between the XhoI and BglII sites. A 3’UTR carrying a mutated sequence in the miR-92a-1-5p seed region was generated by mutagenesis within the pMD18-T vector (Takara, Japan) using a MutanBest Kit (Takara, Japan) and then subcloned into the PGL3-CMV-LUC-MCS vector. The insertions were verified by sequencing. HEK-293T cells were plated in 24-well plates and transfected with either 50 nM miRNA mimic or control RNA along with 100 ng of the PGL3-CMV-LUC-MCS vector. Cells were harvested 48 h after transfection and relative luciferase activity was measured using a Dual-Glo Luciferase Assay Kit (Promega, Madison, WI, USA).

### miR-92a-1-5p overexpression

Recombinant lentiviral vectors carrying the miR-92a-1-5p and *GFP* genes were purchased from Hanheng Biotech (Shanghai, China). K562 cells (2×10^5^ cells/well) were seeded in a 96-well plate and transfected with recombinant adenovirus (100 virus particles/cell). After centrifugation at 1000 × g for 1 h (at room temperature), cells were cultured at 37°C for 48-72 h and selected in medium containing 2 μg/mL puromycin (Gibco). Cellular expression of miR-92a-1-5p and MLKL was determined by qRT-PCR and western blot analysis.

### *in situ* hybridization

LNA-modified, 5’-DIG- and 3’-DIG-labeled probes specific for miR-92a-1-5p, and a negative control probe (miRCURY-LNA detection probes, Exiqon) were used for ISH assays. Probes’ sequences were: miR-92a-1-5p, 5’-CATTGCAACCGATCCCAACCT-3’; and negative control scrambled oligonucleotide sequence, 5’-GTGTAACACGTCTATACGCCCA-3’. ISH was performed using a miRNA ISH Optimization Kit (Exiqon, Woburn, MA) after tissue deparaffinization based on the standard protocol provided by the manufacturer.

### Statistical analysis

Data are presented as the mean ± standard deviation (SD). Differences between groups were analyzed by ANOVA and Student’s t-test. P < 0.05 was considered significant.

### Ethical approval

The Ethics Committee of the First Affiliated Hospital of Zhejiang University (Hangzhou, China) approved the use of human specimens in the current study. Written informed consent was obtained from all patients. All methods and experimental protocols were approved by the First Affiliated Hospital of Zhejiang University.

## Supplementary Material

Supplementary Figure 1

Supplementary Table 1
