# Supplementary material for: Shikonin overcomes drug resistance and induces necroptosis by regulating the miR-92a-1-5p/MLKL axis in chronic myeloid leukemia
Source: Aging (Albany NY). 2020 Sep 14;12(17):17662–80. doi: 10.18632/aging.103844 (PMC7521527; doi:10.18632/aging.103844)
Supplement: Supplementary Figure 1 [file aging-12-103844-s001..pdf]

SUPPLEMENTARY FIGURE

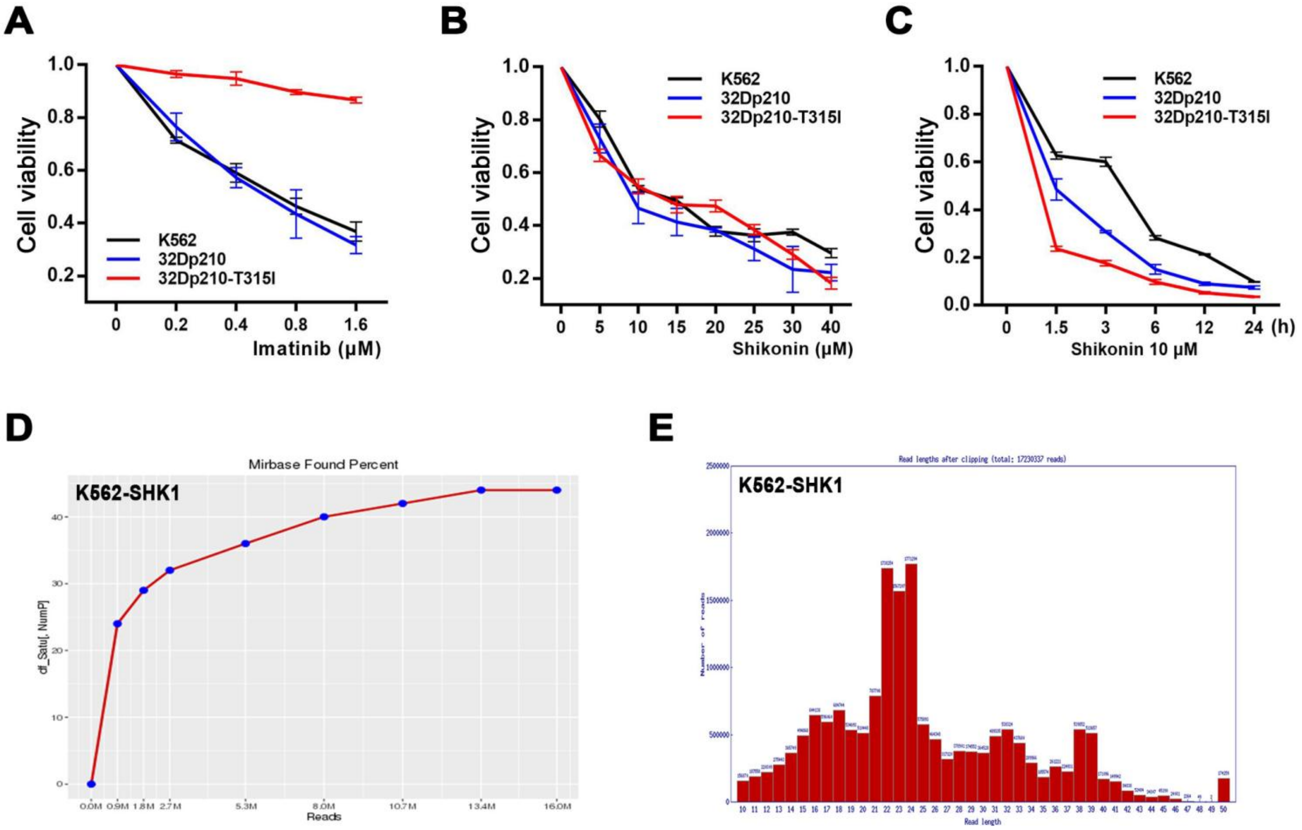

Supplementary Figure 1. (**A**) CML cells (K562, 32Dp210, and 32Dp210-T315I) were treated with various imatinib concentrations (0, 0.2, 0.4, 0.8, or 1.6 μM) for 24 h. Cell survival rates were measured with an MTT assay. (**B**) CML cell lines were cultured with or without different concentrations of shikonin for 3 h. Growth inhibition was assessed with an MTT assay. (**C**) MTT viability assay was performed in CML cells treated with 10 μM shikonin for different times (0, 1.5, 3, 6, 12, or 24 h). (**D**) Saturation analysis of each sample during NGS. (**E**) Read length distribution in each sample after adapter trimming and quality control. All experiments were repeated at least three times.
