# Supplementary material for: Shikonin overcomes drug resistance and induces necroptosis by regulating the miR-92a-1-5p/MLKL axis in chronic myeloid leukemia
Source: Aging (Albany NY). 2020 Sep 14;12(17):17662–80. doi: 10.18632/aging.103844 (PMC7521527; doi:10.18632/aging.103844)
Supplement: Supplementary Table 1 [file aging-12-103844-s002..pdf]

SUPPLEMENTARY TABLE

Supplementary Table 1. Clinical characteristics of patients.

Patients	Sex/age, y	Diagnosis	Prior treatment	Bcr/abl expression	Mutation
1	M/25	Normal	Newly diagnosed	Negative	/
2	M/29	Normal	Newly diagnosed	Negative	/
3	F/29	Normal	Newly diagnosed	Negative	/
4	M/25	Normal	Newly diagnosed	Negative	/
5	F/49	Myeloid sarcoma	Chemotherapy with HA scheme	Negative	/
6	M/22	Amygdalitis	Newly diagnosed	Negative	/
7	F/63	lymphadenectasis	Newly diagnosed	Negative	/
8	M/78	Hemolytic anemia	Newly diagnosed	Negative	/
9	F/34	CML-CP	Newly diagnosed	91%	None
10	M/31	CML-CP	Newly diagnosed	65%	None
11	F/50	CML-CP	Newly diagnosed	95%	None
12	F/46	CML-CP	Newly diagnosed	86%	None
13	M/47	CML-CP	Newly diagnosed	78%	None
14	F/62	CML-CP	Newly diagnosed	29%	None
15	M/62	CML-BC	Imatinib	66%	E453G
16	M/23	CML-BC	Imatinib	76%	None
17	F/51	CML-BC	Imatinib	75%	None
18	F/68	CML-BC	Imatinib	64%	None
19	M/15	CML-BC	Imatinib	121%	T315I, E255K
20	M/24	CML-BC	Imatinib	97%	None
21	M/70	CML-BC	Imatinib	55%	None
